# Classifying Goliath Grouper (*Epinephelus itajara*) Behaviors from a Novel, Multi-Sensor Tag

**DOI:** 10.3390/s21196392

**Published:** 2021-09-24

**Authors:** Lauran R. Brewster, Ali K. Ibrahim, Breanna C. DeGroot, Thomas J. Ostendorf, Hanqi Zhuang, Laurent M. Chérubin, Matthew J. Ajemian

**Affiliations:** 1 Harbor Branch Oceanographic Institute, Florida Atlantic University, Fort Pierce, FL 34946, USA; aibrahim2014@fau.edu (A.K.I.); bdegroot2017@fau.edu (B.C.D.); tostendorf@fau.edu (T.J.O.); lcherubin@fau.edu (L.M.C.); majemian@fau.edu (M.J.A.); 2 Department of Electrical Engineering and Computer Science, Florida Atlantic University, Boca Raton, FL 33431, USA; zhuang@fau.edu

**Keywords:** accelerometer, magnetometer, gyroscope, classification, random forest, support vector machine, deep-learning, bio-logging

## Abstract

Inertial measurement unit sensors (IMU; i.e., accelerometer, gyroscope and magnetometer combinations) are frequently fitted to animals to better understand their activity patterns and energy expenditure. Capable of recording hundreds of data points a second, these sensors can quickly produce large datasets that require methods to automate behavioral classification. Here, we describe behaviors derived from a custom-built multi-sensor bio-logging tag attached to Atlantic Goliath grouper (*Epinephelus itajara*) within a simulated ecosystem. We then compared the performance of two commonly applied machine learning approaches (random forest and support vector machine) to a deep learning approach (convolutional neural network, or CNN) for classifying IMU data from this tag. CNNs are frequently used to recognize activities from IMU data obtained from humans but are less commonly considered for other animals. Thirteen behavioral classes were identified during ethogram development, nine of which were classified. For the conventional machine learning approaches, 187 summary statistics were extracted from the data, including time and frequency domain features. The CNN was fed absolute values obtained from fast Fourier transformations of the raw tri-axial accelerometer, gyroscope and magnetometer channels, with a frequency resolution of 512 data points. Five metrics were used to assess classifier performance; the deep learning approach performed better across all metrics (Sensitivity = 0.962; Specificity = 0.996; *F*_1_-score = 0.962; Matthew’s Correlation Coefficient = 0.959; Cohen’s Kappa = 0.833) than both conventional machine learning approaches. Generally, the random forest performed better than the support vector machine. In some instances, a conventional learning approach yielded a higher performance metric for particular classes (e.g., the random forest had a *F*_1_-score of 0.971 for backward swimming compared to 0.955 for the CNN). Deep learning approaches could potentially improve behavioral classification from IMU data, beyond that obtained from conventional machine learning methods.

## 1. Introduction

The past few decades have seen the development, miniaturization and cost reduction of a variety of sensors that can be attached to animals to monitor their behavior, physiology and environment [1]. Data (archival) loggers are particularly appealing if the device can be retrieved due to their capacity to store large datasets, allowing for high sampling frequencies and thus fine-scale monitoring [2]. Often, sensors are used in tandem to better identify and contextualize behavior. For example, a tri-axial accelerometer can be used to measure body motion and posture in the three orthogonal planes, through dynamic and gravitational forces, respectively. In turn, distinct behaviors corresponding to these waveform signatures can be identified (through direct-observation, i.e., “ground-truthing”) or inferred, which has made them a popular choice for scientists aiming to understand the activity of an animal in the wild. When used in conjunction with sensors that provide information on the body’s angular velocity and rotation—through a gyroscope and magnetometer, respectively—the ability to reconstruct and differentiate behaviors can be improved [3,4,5]. However, with each sensor potentially yielding millions of data points, manually deciphering behaviors from these inertial measurement unit (IMU) data sets is impractical. As such, numerous machine learning (ML) methods have been employed to automate the process of classifying animal-borne sensor output into behavioral classes [6,7,8,9].

Murphy [10] defines ML as “a set of methods that can automatically detect patterns in data, and then use the uncovered patterns to predict future data, or to perform other kinds of decision making under uncertainty”. ML is typically divided into two main types, supervised and unsupervised learning, each with advantages and disadvantages [8]. In supervised learning, a training data set is required whereby the input vector(s) *x* (e.g., sensor channel features) and associated outcome measure/label in vector *y* (e.g., behavior) are known. Once the input vectors can be appropriately mapped to the outcome, the algorithm can be used to make predictions from new input data [11]. This is termed supervised learning, as the outcome label is provided by an “instructor” who tells the ML algorithm what to do. If an animal cannot be housed in captivity for direct observation, or simultaneously fitted with the sensor(s) and a video camera while in situ, building a detailed training set may not be possible. In such instances, unsupervised learning can be implemented. Pre-defined classes are not provided by an instructor (hence “unsupervised learning”), but rather the algorithm finds structure in the data, grouping it based on inherent similarities between input variables [11]. While the terms supervised and unsupervised learning help to categorize some of the methods available, the two concepts are not mutually exclusive and can be used in tandem when labeled data is available for only a portion of the dataset (e.g., semi-supervised, multi-instance learning). 

**Figure 1:**
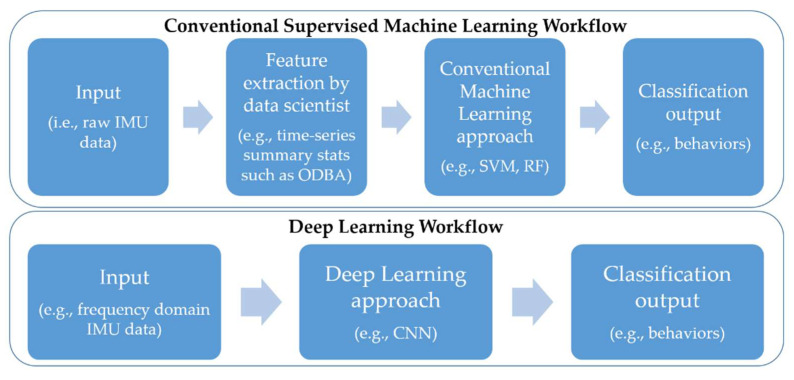
Simplified schematic showing the workflow of conventional machine learning approaches versus deep learning approaches. IMU = inertial measurement unit, ODBA = overall dynamic body acceleration, SVM = support vector machine, RF = random forest, CNN = Convolutional Neural Network.

Recently, deep learning approaches have become popular for modeling high-level data in areas such as image classification [12], text classification [13], medical data classification [14] and acoustic sound classification [15]. Unlike supervised machine learning approaches, deep learning is a form of ML that does not require a manual extraction of features for training the model but instead can be fed raw data (Figure 1). Its development was driven by the challenges faced by conventional ML algorithms including the inability to generalize well to new data, particularly when working with high-dimensional data and the computational power required to do so.

Various deep learning approaches have been applied to accelerometer data for human activity classification including convolutional neural networks (CNNs), long short-term memory (LSTM) and a combination of the two [16,17,18,19,20,21,22,23,24]. Aviléz-Cruz et al. [19] proposed a deep learning model that achieved 100% accuracy across six activities, compared with 98% and 96% for the two most competitive conventional ML approaches (Hidden Markov Model and support vector machine, SVM, respectively). The model had three CNNs working in parallel, all receiving the same input signal from a tri-axial accelerometer and gyroscope. The feature maps of the three CNNs were flattened and concatenated before being passed into a fully connected layer and finally an output layer with a Softmax activation (a function that converts the numbers/logits generated by the last fully connected layer, into a probability that an observation belongs to each potential class [25]). Other studies demonstrate the relevance of using LSTM networks for human activity recognition [17,20,21,22,23]. Lastly, a few studies have suggested augmenting CNNs with LSTM layers [26]. For example, Karim et al. [26] proposed a model architecture in which a three-layer CNN and an LSTM layer extract features from sensor data in parallel. The resulting feature vectors are then concatenated and passed into a Softmax classification layer. Although deep learning can yield improved classifier performance over conventional ML methods, it has been sparsely applied for animal behavior detection from IMU data [8]. 

Within the realm of marine fishes, IMU sensors have been widely applied to highly mobile species including sharks [27,28,29], Atlantic bluefin tuna (*Thunnus thynnus*) [30], dolphin fish (*Coryphaena hippurus*) [31] and amberjack (*Seriola lalandi*) [32], providing insight into biomechanics, activity patterns, energy expenditure, diving and spawning behavior. However, application of IMUs to more sedentary species that persist predominantly over highly complex structures, such as natural and artificial reefs, are rarer. These species, for example grouper, can be expected to engage in different behaviors to that of highly mobile species and present a different activity budget.

Groupers (family *Epinephelidae*) are comprised of more than 160 species of commercially and recreationally important fishes that inhabit coastal areas of the tropics and subtropics [33]. This family of long-lived fishes shares life history traits that make them particularly vulnerable to overfishing, including: late sexual maturity, protogyny, and the formation of spawning aggregations [34,35,36,37]. The Atlantic Goliath Grouper (*Epinephelus itajara* Lichtenstein 1822; hereafter referred to as Goliath grouper) is one of the largest grouper species, capable of attaining lengths of 2.5 m and exceeding 400 kg [38]. The species ranges from North Carolina to Brazil and throughout the Gulf of Mexico [39]. Much of our understanding of Goliath grouper behavior has been learned from divers, from underwater video footage, and observing animals in captivity (e.g., feeding kinematics [40], abundance [41]). Passive acoustic monitoring of sound production (e.g., associated with spawning behavior) [42,43] and modest acoustic telemetry work has provided some insight into site fidelity and coarse horizontal and vertical movement [44]. To date, no studies have documented the fine-scale behavior of this species. IMUs provide the opportunity to learn about fine-scale Goliath grouper activity patterns over a range of temporal scales, and the energetic implications. Additionally, IMUs can yield insight into, inter alia, mating behavior, habitat selection and responses to environmental variables [45,46].

Accelerometer transmitters have been used to determine activity levels (active versus inactive) [47] and feeding behavior [48] of captive red-spotted groupers (*Epinephelus akaara*). An accelerometer-gyroscope data logger was used to identify feeding and escape response behavior of captive White-streaked grouper (*Epinephelus ongus*) [3]. In both studies, behaviors were validated using underwater video cameras situated in the tank. To our knowledge, no studies have used IMU sensors to elucidate the behavior of grouper species at liberty. However, as one of the largest grouper species, Goliath grouper can be equipped with multi-sensor tags that include a video camera for validation of IMU data obtained from individuals in the wild. 

The goals of this study were to: (a) obtain ground-truthed body movement data from a custom-made tag fitted to Goliath grouper, which could be used to develop a behavioral classifier; (b) develop two conventional ML approaches, using handcrafted features, to classify behavior from the tag data; (c) design a deep learning approach using CNN and frequency representations of IMU data; and (d) compare the performance of the conventional ML approaches to the deep learning approach to determine the preferred method for identifying and studying behaviors from animals at liberty. Knowledge of the fine-scale activity of these animals can help us understand the ecology of this species, a key research need highlighted by the International Union for the Conservation of Nature [39].

## 2. Materials and Methods

### 2.1. Study Site and Capture

**Figure 2:**
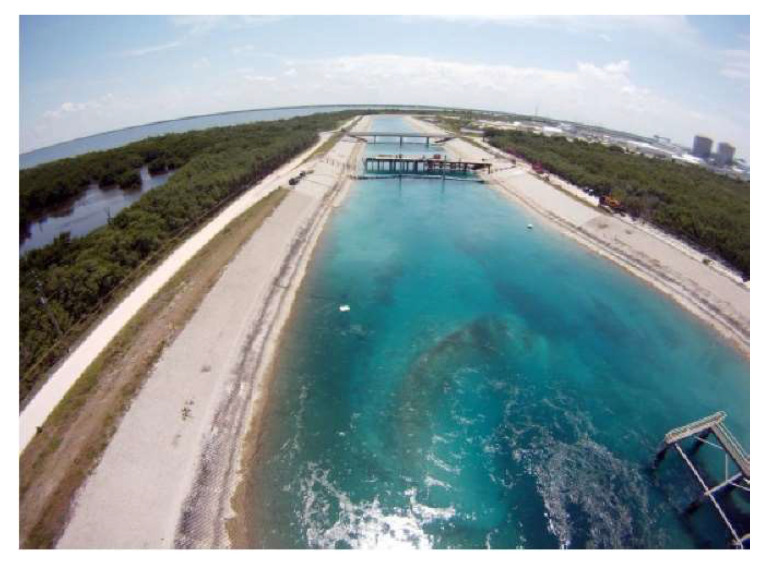
The study site: the entrainment canal at St. Lucie nuclear power plant facility located on south Hutchinson Island, Florida. Permanent mesh barriers are located underneath each bridge, keeping marine fauna in the entrainment at the forefront of the photograph. Photo credit: Serge Aucoin.

Goliath groupers were captured at the St. Lucie nuclear power plant facility located on south Hutchinson Island, Florida (27.20° N, 80.14° W). The power plant draws in seawater from approximately 365 m offshore in the Northwest Atlantic Ocean to help cool the nuclear reactors. Water is drawn in at a rate of ~one million gallons per minute, through three large diameter pipes (3.7–4.9 m), and exits into a 1500 m intake canal [49,50]. Permanent mesh barriers span the width of the canal to prevent marine organisms that have travelled through the pipes from entering the plant. The first barrier is situated ~160 m from the pipes, creating an entrainment area ~160 m long x 80 m wide, max depth ~5 m (Figure 2). This entrainment provides a semi-natural environment for animals, including Goliath grouper, to inhabit.

In the entrainment, Goliath grouper were caught using a hand-reel with 250 lb. monofilament and a 16/0 circle hook with the barb filed back. Bait was primarily thawed striped mullet (*Mugil cephalus*). Once reeled in, the individual was brought onboard a low gunnel 14’ skiff and transported the short distance to a ramp adjacent to the pipes, where it was placed in a sling and a hose was inserted into the buccal cavity to actively pump water over the gills during handling. Prior to fitting the bio-logging tag, morphometric measurements including total length and girth were recorded and the animal was fitted with a plastic tipped dart tag at the base of the dorsal spines for future identification (Table 1). All efforts were made to minimize animal pain and suffering during collection and all activities followed approved animal use protocols (FAU AUP #A18-28; ACURO #DARPA-7374.02).

### 2.2. Tag Attachment

**Figure 3:**
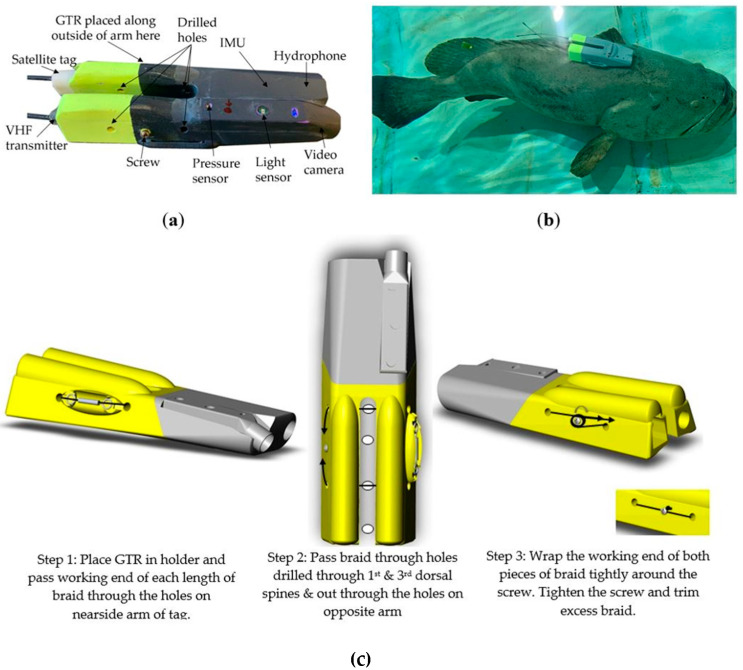
Custom-designed bio-logging tag used on Goliath grouper: (**a**) the components of the tag; (**b**) attachment location of the tag; (**c**) the tag attachment process GTR = galvanic timed release.

We designed a custom multi-sensor tag with Customized Animal Tracking Solutions for use on Goliath grouper, measuring 24.5(L) × 9(W) × 5(D) cm (Figure 3). The tag comprised a tri-axial accelerometer, gyroscope and magnetometer (hereinafter collectively referred to as IMU), a temperature, pressure and light sensor, video camera (1920 × 1080 resolution) and hydrophone (HTI-96-Min Series with a sensitivity of −201 dB re 1 μPa), all mounted in the anterior portion of the tag. Hydrophone data were not used in this case given our interest in classifying behavior from kinematic variables. The posterior end of the tag consisted of two positively buoyant “arms” that facilitate tag ascent to the surface once it released from the fish. This portion also housed a VHF transmitter and satellite transmitter to aid in relocating the device so the IMU and video data could be downloaded. The custom tags were programmed to record acceleration data at either 50 or 200 Hz, gyroscope and magnetometer data at 50 Hz, and pressure and temp at 1 Hz. Tags were programmed to commence recording IMU and video data at either 7 or 8 a.m. (depending on sunrise time) the morning after the fish was released. The delay in video recording allowed for post-release recovery (17.0–22.5 h depending on capture time), increasing the chances of capturing normal behavior as the tag was limited to recording ~10 h of video footage. 

The tag was positioned atop the fish with the camera facing anteriorly and arms situated around the dorsal spines (Figure 3b). A three-day tropical galvanic timed release (model C6) was positioned parallel to the outside edge of one arm with 80 lb. microfilament braided line (~30 cm long) placed in either end of the barrel and held in place with the galvanic timed release eyelets. Two holes were drilled through each arm of the tag, one on either side of the galvanic timed release barrel, so that the working end of each length of braid could pass through both arms. A small hole (1/32” = 0.79 mm) was also drilled through the first and third dorsal spines so that the working ends of the braid could each pass through a spine in between the arms. On the opposite side of the tag to the galvanic timed release barrel, the working ends were wrapped clockwise around a screw embedded into the float material. The screw was then tightened to pull the braid taut and secure the tag to the fish (Figure 3c). The tag released from the fish after the galvanic timed release corroded and the ends of the braid embedded in the barrel became free to pull through the spines as the tag floated to the surface. Tags were retrieved from the entrainment canal by on site personnel and the data downloaded using CATS-Diary software (version 6.1.35).

### 2.3. Data Analysis

#### 2.3.1. Ethogram and Feature Extraction

**Figure 4:**
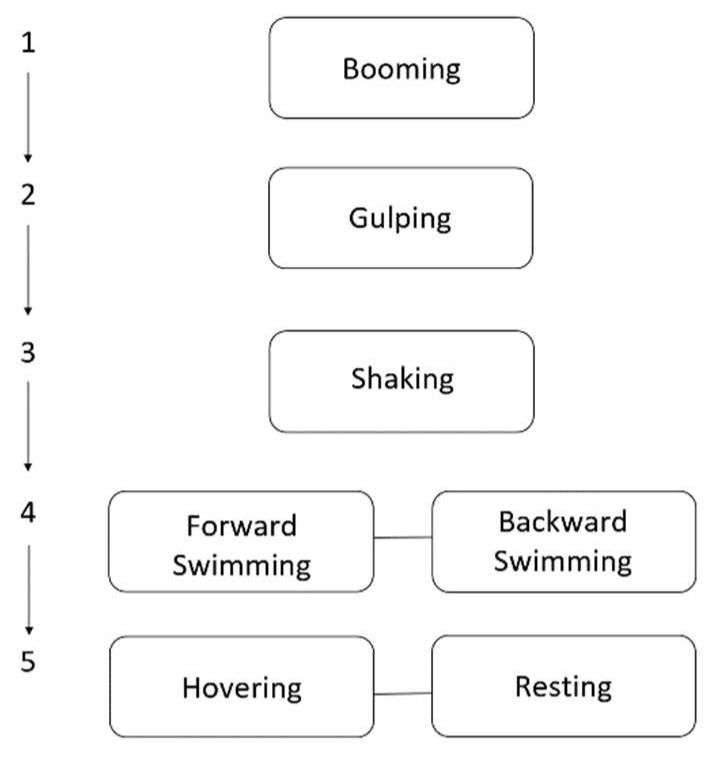
Hierarchy used to label behavioral classes when an animal was performing simultaneous behaviors. For example, if an individual was both forward swimming and booming, those data points would be labeled as booming.

An ethogram of behaviors (Table 2) was developed using video footage from the tag across six deployments (Table 1) where the water visibility was sufficient to yield clear recordings (See Video S1 in Supplementary Materials). As individuals were able to conduct multiple behaviors simultaneously (e.g., hovering and booming or swimming and turning), a labeling hierarchy was developed for assigning data to a single class in those instances (Figure 4).

Feature data were calculated from the IMU data over 1 s intervals and each second of data was assigned a behavioral class. A total of 187 features were calculated for each deployment including summary statistics from each orthogonal plane of the accelerometer, magnetometer and gyroscope sensors. The summary statistics included time and frequency domain features. Time domain summary statistics included average, standard deviation, minimum, maximum, median, skewness, kurtosis, median absolute deviation, inverse covariance, and interquartile range. Summary statistics were also calculated for overall dynamic body acceleration (ODBA) [6,7,8,51,52]. The accelerometer records total acceleration which comprises the gravitational component of acceleration (which reflects tag orientation, and thus animal posture, in relation to the earth’s gravitational pull) and dynamic acceleration caused by the animals’ body movement. The gravitational component of acceleration was calculated by applying a 3 s running mean to the total acceleration and subtracting it to leave dynamic acceleration. ODBA was then calculated as the sum of the absolute dynamic axes values [53]. Additional time domain variables included signal magnitude area (sum of the absolute raw acceleration axes), *q* (calculated for each IMU sensor as the square-root of the sum-of-squares of the three axes), the circular variances of the inclination and azimuth of each *q*, pairwise correlations between the accelerometer axes [6,52] and vertical velocity. All time domain features were calculated in R Core Team (2020) [54]. Frequency domain features included power, mean, standard deviation, median, minimum, maximum, entropy and energy calculated from the spectrum for each orthogonal plane of the accelerometer, magnetometer and gyroscope sensors [55]. Frequency domain features were calculated in MATLAB 2019a.

#### 2.3.2. Conventional Machine Learning Models

Two supervised ML algorithms—a random forest (RF) and a SVM—were built using MATLAB 2019a. Both algorithms have been commonly employed to recognize behavior from acceleration data obtained from numerous species [6,7,56,57,58]. Ensemble classifiers, such as RFs, combine predictions from multiple base estimators to make a more robust model. In the case of RF, many independent, un-pruned classification trees are produced with each tree predicting a class for the given event. To minimize overfitting, two levels of randomness are incorporated: (1) a random subsample of data (62.3%) are used to generate every tree and (2) at each tree node, a random subset of predictor variables (m) is selected to encourage tree diversity. The final prediction is usually selected as the class with the majority vote from all the trees [59]. As a random subsample of the full dataset is used to build each tree (a process known as bootstrap aggregation or “bagging”), RFs are considered bagging ensemble classifiers. SVM, a supervised machine learning method, aims to design an optimal hyperplane that separates the input features into two classes for binary classification. The input data to SVM is mapped into high-dimensional feature space by using a kernel function. In this study, the RF was built using 200 trees and the SVM was constructed using a Gaussian radial kernel function.

#### 2.3.3. Deep Learning Approach

**Figure 5:**
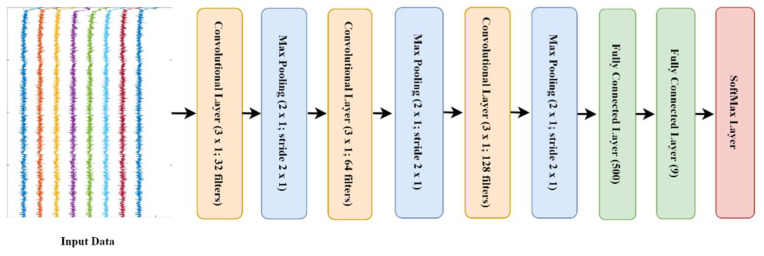
Schematic of convolutional neural network model.

For the deep learning approach, we developed a CNN to work with the 1-dimensional spectrum of each of the three accelerometer, magnetometer and gyroscope axes. The CNN comprised three convolutional layers—with one-dimensional kernel size (3 × 1)—with each layer followed by a maxpooling layer to reduce the dimensionality of the convolutional layer and control overfitting. These convolutional and maxpooling layers extract high-level features from the data which are then used as the input into the fully connected layers for classification. The final maxpooling layer was followed by a fully connected layer with 500 nodes, a dropout layer with 0.25 probability and a fully connected layer with Softmax activation that ensures the output predictions across all classes sum to one (Figure 5). The input to the model consists of nine channels of frequency representations, one for each IMU axis. Each channel was converted to Fourier transform with *NFFT* = 512, and the absolute value computed. The input size of the network was 256 × 9 with each column representing the frequency transformation of each axis. To find the relationship between input data *X*, and output class Z, we have to find:

Z = *F*(*X*/*λ*) (1)

where *F* is a non-linear function which maps the input matrix *X* to output vector ***z***, and *λ_k_* is a collection of weights *W_k_* and biases *B_k_* at layer *k*, and is the collection of all weights and biases in the network. We can express this relationship as:

***z** = F(X/λ) = f_l_(…f_2_(f_1_(X/λ_1_)/λ_2_))* (2)

where each small function *f_l_(*./*λ_l_)* is referred to as a layer of the CNN. For this neural network, we used *l* = 9. Layers one, three and five are convolutional layers, expressed as:

*Out_l_* = *f_l_*(*X_l_*/*λ_l_*) = *h*(*W_l_∗X_l_ + B_l_*), *λ_l_* = [*W_l_*, *B_l_*] (3)

where *X_l_* is the input to the last layer of the network, *h* is an activation function (in our case we used a Rectified Linear Unit (ReLU) as the activation function).

The proposed CNN architecture is parameterized as follows:

*l*_1_: 32 kernels of size (3 × 1) which work on each frequency transformation of the input data, this is followed by maxpooling of pool size [2, 1] with stride two.
*l*_3_: 64 kernels of size (3 × 1) which work on each frequency transformation of the input data, this is followed by maxpooling of pool size [2, 1] with stride two.
*l*_5_: 128 kernels of size (3 × 1) which work on each frequency transformation of the input data, this is followed by maxpooling of pool size [2, 1] with stride two.
*l*_7_: a fully connected layer with 500 nodes followed by drop out layer with probability 0.25.
*l*_9_: a fully connected layer with 9 nodes followed by Softmax activation layer.

#### 2.3.4. Data Augmentation

Behavioral classification is predisposed to unequal class sizes because animals do not partition their time equally between activities. Data augmentation can be used to increase the number of events in minority classes [60] and can be viewed as an injection of prior knowledge about the invariant properties of the IMU data against certain transformations. Augmented data can also cover unexplored input space, prevent overfitting, and improve the generalization ability of a deep learning model, with many data augmentation methods available (e.g., GAN network, scaling, rotation and data oversampling) [61]. In this study, we applied three data augmentation techniques that are commonly applied to acceleration data [60,62,63]:

*Jittering*: One of the most effective data augmentation methods. Jittering adds normally distributed noise to the IMU data. Jittering can be defined as:

***x** = x_1_ + e_1_, x_2_ + e_2_, …, x_N_ + e_N_* (4)

where ***x*** *=* [*x*_1_, *x*_2_, *…*, *x_N_*]*^T^* is the vector of the actual data points and ***e*** *=* [*e*_1_, *e*_2_, *…*, *e_N_*]*^T^* is the vector of the added points. e is the normal distribution noise added to the data points and *e_i_* *∼ N*(*0*, *σ^2^*), where *σ* is a hyper-parameter of range [0.01, 0.2].

*Magnitude scaling*: Magnitude scaling changes the global magnitude of the IMU data by a randomly selected scalar value. Scaling is a multiplication of the entire dataset as follows:

*X* = [*γx_1_,γx_2_, …,γx_N_*]*^T^* (5)

The scaling parameter *γ* can be determined by normal distribution *γ* ∼ *N*(1, *σ*^2^), where *σ* is a hyper-parameter.

*Magnitude warping*: Magnitude warping warps a signal’s magnitude by a smoothed curve as follows:

*X* = *β*_1_
*x*_1_,*β*_2_
*x*_2_, …,*β_N_ x_N_* (6)

where *β*_1_, *β*_2_, *…*, *β_N_* is a sequence interpolated from cubic spline *S*(*k*) with *k = k*_1_, *k*_2_, *…*, *k_l_*. Each knot *k_i_* is given a distribution *γ* ∼ *N*(1, *σ*^2^), where the number of knots and the standard deviation σ are hyper-parameters. The idea behind magnitude warping is that small fluctuations in the data can be added by increasing or decreasing random regions in the IMU data.

#### 2.3.5. Performance Measures

To evaluate the classifiers, we retained 20% of the ground-truthed data for testing via five-fold validation. We adopted five performance measures including: sensitivity (recall), specificity, *F*_1_-score, Matthews Correlation Coefficient (MCC) [64] and Kappa. These metrics were calculated for each class and for the classifier overall. Sensitivity determines the proportion of events that were correctly classified; specificity indicates the proportion of events that are correctly identified as not belonging to a class. To compute these measurements, the true positive (*TP*), true negative (*TN*), false positive (*FP*), and false negative (*FN*) were extracted for each class from the confusion matrices. Sensitivity can be computed using the following formula:

(7) $$\mathrm{Sensitivity}=\frac{TP}{TP+FN}.$$

Specificity or true negative rate is calculated as:

(8) $$\mathrm{Specificity}=\frac{TN}{TN+FP}.$$

*F*_1_-score is the harmonic mean of precision and sensitivity. Precision represents the fraction of correctly identified classes (i.e., sensitivity) against all predicted classes and is calculated as:

(9) $$\mathrm{Precision}=\frac{TP}{TP+FP}.$$

Thus, the *F*_1_-score is calculated as:

(10) $$F_{1}=\frac{2TP}{\left(2TP+FN+FP\right)}.$$

Sensitivity, specificity and the *F*_1_-score are presented as a value between 0 and 1, where a value closer to 1 indicates good classification performance. 

The MCC can be calculated by the following equation:

(11) $$\mathrm{MCC}=\frac{TP\;x\;TN-FP\times FN}{\sqrt{\left(TP+FP\right)\left(TP+FN\right)\left(TN+FP\right)\left(TN+FN\right)}}.$$

The Kappa statistic provides a quantitative measure of how well the classifier agrees with the ground-truth data while accounting for agreement that would be expected to occur by chance [65] (i.e., than a classifier that guesses the class based on class frequency). Kappa is capable of handling both multi-class and imbalanced class problems [66] and can be defined as:

(12) $$K=\frac{P_{o}-P_{e}}{1-P_{e}}$$

where *P_o_* is the observed agreement and *P_e_* is the expected agreement. The value of *K* between 0.4 and 0.6 is considered as moderate, between 0.61 and 0.80 as substantial and between 0.81 and 1 as almost perfect agreement [65].

For each metric (except Kappa), overall performance was calculated as the mean of the metric values determined for each class. Overall Kappa performance was calculated using Equations (13)–(15) as follows:

(13) $$P_{ex}=\frac{\left(P_{x}^{*}\left(TP_{x}+FP_{x}\right)\right)+\left(N_{x}^{*}\left(FN_{x}+TN_{x}\right)\right)}{{\left(TP_{x}+TN_{x}+FP_{x}+FN_{x}\right)}^{2}}$$

where *P_x_* is the sum of all positive classifications, *TP_x_* is the sum of all *TP*s, *FP_x_* is the sum of all *FP*s, *N_x_* is the sum of all negative classifications, *TN_x_* is the sum of all *TN*s and *FN_x_* is the sum of all FNs.

(14) $$\mathrm{kappa}=\left(\left[\frac{\left(P_{ox}-P_{ex}\right)}{1-P_{ex}}\;,\;\frac{P_{ex}-P_{ox}}{1-P_{ex}}\right]\right)$$

where $P_{ox}$ is the sum of accuracy values for all classes. Finally:

(15) Overall Kappa Performance=max(kappa)

## 3. Results

For this study, data were collected from six fish. Using a three-day galvanic timed release, the average tag retention time was 68.5 h (SD = 6.7 h; Table 1). This allowed ample time for the tag battery to fully deplete prior to releasing from the animal and thus maximized the amount of IMU data that could be obtained from each deployment. The video footage revealed that tagged individuals regularly interacted with non-tagged animals within the entrainment and appeared to exhibit similar behavior.

### 3.1. Ethogram Development

Each second of IMU data was assigned one of 13 behavioral classes identified from the animal-borne video footage; 52.98 h of IMU data were labeled. The time each fish engaged in a behavior varied and not all individuals exhibited every behavior (Table 3). The most common behaviors were hovering, forward swimming and resting. Four of the 13 identified classes were omitted from the classifier because we were unable to gather enough data to create a robust training dataset for that class (i.e., feeding and rolling) and/or the behaviors were not performed by most individuals (i.e., burst swimming and gliding). Three animals exhibited burst swimming, yielding a combined total of 337 s of data for this class. Gliding usually occurred after a burst swim and was exhibited only by two of the three animals that burst swam. Only one animal fed while the tag was fitted and recording video, yielding 58 s of feeding behavior. Rolling was documented for five of the six animals, but these events were infrequent and brief, so not allowing for sufficient data accumulation to develop this class.

### 3.2. Classifier Performance

**Figure 6:**
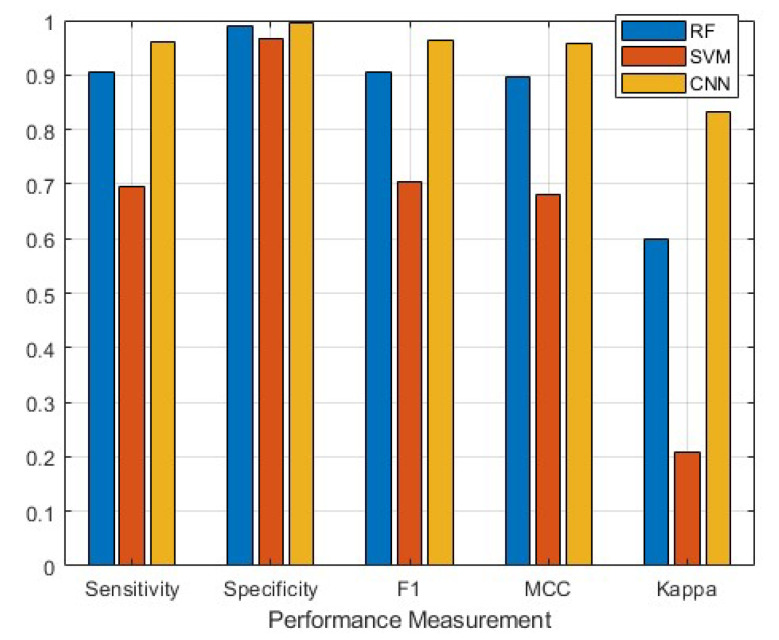
A comparison of the overall performance metrics for each approach: random forest (RF), support vector machine (SVM) and convolutional neural network (CNN)**.** MCC is the Matthew’s Correlation Coefficient.

The deep learning approach produced the highest overall values across each performance metric while the SVM produced the lowest (Figure 6). The CNN was the only method to attain a kappa value >0.81, indicating almost perfect agreement between the classifier and the labeled data (Table 4). Conversely, the SVM obtained κ = 0.21, suggesting poor agreement between the classifier and labeled data (Table 4). The RF achieved κ = 0.60, indicating moderate agreement (Table 4). All models obtained an overall specificity ≥0.97, with models performing better in terms of specificity than sensitivity (0.70–0.96; Table 5 and Table 6; Figure 6). 

However, the CNN classification did not rank best for all behaviors. For example, the RF obtained a higher specificity, *F*_1_-score and MCC for backward swimming than the CNN (Table 6, Table 7 and Table 8). The RF also obtained a higher specificity for turning (1.0 versus 0.99 for CNN; Table 6). Kappa was the only performance metric that indicated more variable performance between methods on a class-by-class basis (Table 4). The CNN performed better than either conventional ML approach for four of the nine classes (forward and backward swimming, listing and gulping) but scored lowest on three of the classes (booming = 0.86, i.e., almost perfect agreement; shaking = 0.75, i.e., substantial agreement; turning = 0.45, i.e., moderate agreement). 

Of the conventional ML algorithms, RF performed better overall than the SVM for each performance metric (Table 4, Table 5, Table 6, Table 7 and Table 8, Figure 6). However, the SVM achieved higher sensitivity than the RF for the forward swim class (0.83 and 0.76 respectively) and higher kappa values for resting, hovering, booming and turning than either of the other methods (Table 4 and Table 5).

**Figure 7:**
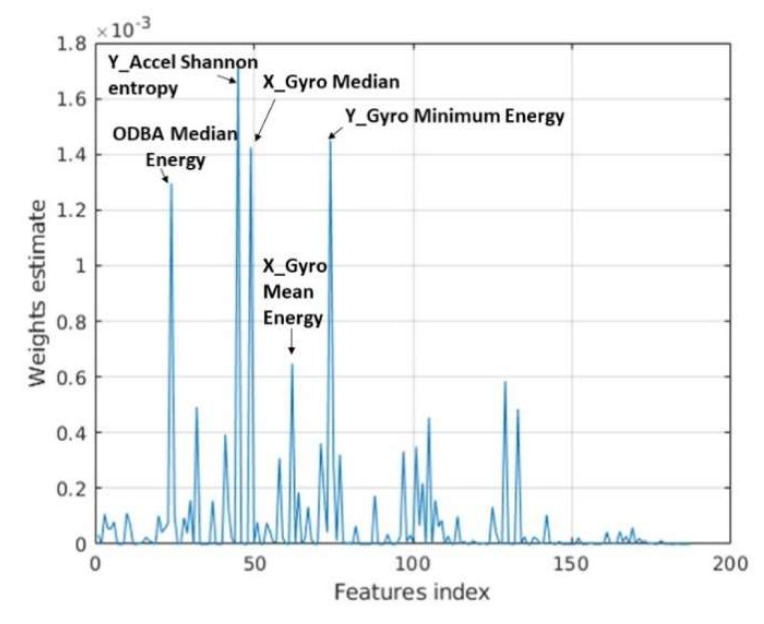
Estimation of feature importance for the random forest (RF) with the five most important features indicated. Note X_Gyro Median is the only time-series feature, while the rest are frequency domain features.

The importance of each feature provided to a RF can be determined by assessing the node risk (i.e., change in node impurity weighted by the node probability) associated with splitting the data using each feature. The top five most important features were Shannon entropy for Y-axis acceleration, with weight = 1.7 × 10^−3^, followed by minimum energy (1.47 × 10^−3^) for Y-axis gyroscope, the median from the X-axis gyroscope (1.44 × 10^−3^), median energy from ODBA (1.3 × 10^−3^) and mean energy from the X-axis gyroscope (0.6 × 10^−3^; Figure 7). 

## 4. Discussion

The aim of this study was to develop and assess the performance of two conventional machine learning methods and a deep learning method for classifying IMU data obtained from Goliath grouper into behavioral classes. Prerequisites to achieving this were the development of a retrievable custom-made tag that recorded IMU data and video concurrently (for ground-truthing) and establishing a robust attachment method. We chose our dorsal spine attachment method as it conferred the following benefits: it was minimally invasive (compared to other tag attachment methods, e.g., drilling through the dorsal musculature [3]), no attachment materials were left in/on the individual when the tag detached, and it resulted in good tag stability on fish > ~1.3 m total length. Tag stability is imperative to the IMU recording data reflective of body movement and ensuring behaviors are discernable from the data between deployments. Smaller fish tended to have narrower spines that did not sufficiently fill the gap between the arms of the tag, resulting in a less stable attachment. A similar tag design and attachment technique to that used here should be applicable to other morphologically similar species such as the Pacific analogs, *Epinephelus tukula*. As sensors, cameras and batteries continue to miniaturize there may be potential for a reduction in overall tag size, perhaps making it applicable for use with smaller species with conservation concerns (e.g., Nassau Grouper, *Epinephelus striatus*).

The tag captured a variety of behaviors, but the activity budget was dominated by hovering and/or resting for all but one individual (Fish 5) that spent 70% of its time swimming. These activity budget patterns may periodically shift to include more activity for individuals at liberty, particularly as Goliath grouper are thought to move to site-specific aggregations during the spawning season [43,67,68]. With low-movement (and thus low-energy) behaviors dominating the activity budget in this study, and the tag only recording video during daylight hours, it is perhaps not surprising that feeding events were infrequent and/or not seen. Goliath grouper are considered opportunistic predators, but feeding was only captured once during the study when fish four consumed a black margate (*Anisotremus surinamensis*). Consequently, we did not obtain enough data to develop a feeding class. Moreover, a study by Collins and Motta (2017) described how Goliath grouper modulate their feeding behavior depending on prey type [40], and thus feeding would likely warrant two classes: suction and ram feeding. When targeting slow-moving or benthic prey, which comprise most Goliath grouper prey items, they employ suction feeding. This involves a slow approach, potentially stopping in front of the prey before it is rapidly sucked into the mouth. When targeting more mobile prey, Goliath grouper typically employ ram feeding, which is characterized by faster capture that includes quicker approaches and wider gapes [40]. Thus, to appropriately classify feeding behavior from IMU data for this species, more data must be collected in future studies. This could be achieved using IMUs that record for longer and are fitted to captive Goliath grouper that can be directly observed/videoed, or from continued deployment of these custom tags to wild individuals.

Using the three learning approaches, we classified nine of the 13 behaviors identified as part of ethogram development. The CNN performed better overall than either conventional ML method according to each of the five metrics calculated. This may be attributable to both the number of features and type of data used as the input to the CNN. The CNN had 36,864 feature maps used as input to the fully connected layer versus 187-handcraft features—spanning the time-series and frequency domain—for the conventional ML approaches. The CNN was developed solely from frequency domain data for each tri-axial IMU sensor and is designed to identify and extract the features (which often have no meaningful interpretation outside of their application) most useful to the classification task. The feature importance plot obtained from the RF indicated four of the five most important features were from the frequency domain (Shannon entropy, minimum, median and mean energy; Figure 7). Therefore, the CNN not only had more features to train from but may have detected important features from the frequency domain that were not extracted as handcraft features for the conventional ML approaches.

Both RF and SVMs are commonly employed to classify IMU data into behaviors. In a study investigating the performance of eight conventional machine learning methods classifying acceleration data into behavioral classes for Port Jackson sharks (*Heterodontus portusjacksoni*), the SVM and RF performed best, using 2 s epochs for labeling the data. The two methods obtained equal overall accuracy (89%) but the SVM achieved superior performance for fine-scale behaviors such as chewing [7]. Conversely, RFs performed better than SVMs for classifying acceleration data obtained from Griffon vultures (*Gyps fulvus*) into seven behaviors [6]. In our study, the RF performed better overall and achieved higher *F*_1_-scores for each class than the SVM. This indicates the importance of model comparison when determining which classifier to use to make predictions from a dataset. No single conventional machine learning algorithm consistently performs best for classifying IMU data into behavioral classes and will be dependent upon factors such as training dataset size, linearity of the data, number of classes and the extent of kinematic similarities between classes (e.g., resting and hovering).

An important consideration when selecting a classifier is whether the researcher is more concerned with identifying a particular behavior or determining overall activity patterns. A need to identify each instance of a particular behavior would require high sensitivity (preferably coupled with good specificity) for that class, which in turn may influence the choice of classifier. The SVM had a marginally higher sensitivity for forward swimming (0.8251) than that obtained by the CNN and RF (0.8007 and 0.7631 respectively). However, it obtained much lower sensitivity values for all other behaviors, including booming (SVM = 0.3282, RF = 0.8733, CNN = 1.000). Goliath grouper produce sound (i.e., “booming”) as part of courtship, spawning and agonistic behavior and is therefore a behavior of particular interest [42]. Passive acoustics can be used to remotely monitor these booms and have been used to determine the relative abundance of soniferous fishes at spawning aggregation sites [42,69]. However, a limitation of using passive acoustics is the inability to approximate how many fish are contributing to sound production. The CNN method developed here robustly classified “booming” behavior from the IMU data and provides a means to determine sound production at the individual level; as such, it may serve as a complementary method to passive acoustic monitoring.

The CNN developed in this study has numerous practical applications for understanding the behavioral ecology of Goliath grouper. IMU sensors are capable of recording data over ever-increasing durations. These tools, coupled with the CNN classifier developed here, present the opportunity to quantify how the activity budget of wild Goliath grouper may differ: temporally (e.g., diel and seasonal patterns), between habitat types (e.g., artificial versus natural reefs) and between pristine habitats and those that are heavily impacted by anthropogenic activity (e.g., fishing, diving, boat traffic). For example, a study that applied accelerometers to red snapper (*Lutjanus campechanus*) found them to be more active over artificial structures (i.e., shipwrecks and submerged oil platform jackets) than on natural reefs, suggesting there may be differences in the functional role of these habitats for red snapper [70]. The same study also documented higher activity levels at night and during the summer. However, without video footage or a behavioral classifier to interpret the acceleration data, the reasons for these differences remain unclear [70]. Other acceleration-based studies have documented impacts of anthropogenic activities on fish behavior, such as impacts of provisioning sites on activity levels of whitetip reef sharks (*Triaenodon obesus*) [71] and dam construction on Chinese sturgeon (*Acipenser sinensis*) swimming behavior [72]. Furthermore, Goliath grouper are targeted for catch-and-release fishing and caught as incidental bycatch by fishermen targeting other reef fishes [73], but little is known about their post-release recovery. The CNN developed herein provides a means to determine if and how the activity budget changes after capture, and how long it may take for an individual to resume normal behavior [74,75].

Custom-made tags such as the one presented here provide an opportunity to document interactions with humans. Stakeholder interactions with Goliath grouper can directly influence their stance on whether Florida should re-open the fishery [73]. Spear fishers claim increased negative encounters with Goliath grouper, while commercial fishermen argue Goliath grouper are impacting their ability to land valuable snapper/grouper species as they presumably depredate their catch [73,76]. Conversely, many recreational dive companies and divers oppose the fishery, with out-of-state divers willing to pay ~336 USD to dive at a Goliath grouper spawning aggregation site [77]. These customized tags can thus help quantify the frequency of these interactions and help make more informed management decisions. Additionally, while not used in this study given the focus on body movement classification, the hydrophone component of the tag could be used to track boat traffic within the vicinity of the fish, as others have done recently with monitoring fishing activity on artificial reef sites [78].

Behavioral classification from animal-borne IMU tags is typically completed once the tag is recovered and the raw data can be downloaded. However, real-time behavioral monitoring requires data transmission from the tag to a nearby receiver. In this case, either the raw data must be transmitted from the tag and be classified onboard the receiver, or the classification occurs onboard the tag and the class prediction is transmitted. A study by le Roux et al. [79] indicated that behavioral classification onboard the tag (using linear discriminant analysis) and transmission reduced the tag’s battery consumption 27-fold compared to transmitting the raw data. This can lead to a substantial increase in the time a tag functions while on the animal, providing obvious benefits (e.g., reducing how often an animal needs to be recaptured if continuous monitoring is required, increased ability to capture rare events, etc.). Alternatively, on-animal classification and storage of the behavior, in favor of storing all the raw data, led to a 469-fold reduction in memory use and a 1.3% increase in power consumption [79]. However, the primary limitation of deep learning is the computational power required, which may prove problematic for on-animal classification where a larger battery, and thus bigger tag would be required. In such instances, a conventional machine learning approach may be more practical.

Overall, our study describes a novel multi-sensor tag with a reliable attachment method to a large reef fish that can be applied to analogous species around the world. Furthermore, analyses of behaviors revealed from the tag indicates better performance of a deep learning approach at classifying IMU data into behaviors compared to two commonly employed conventional ML approaches. The authors recommend that researchers looking to optimize classification of animal-borne IMU data into behavioral classes more regularly consider deep learning approaches alongside conventional ML approaches when developing and selecting a classifier.

## Figures and Tables

**Table 1:** Summary data for goliath grouper deployments at the St. Lucie nuclear power plant facility.

Deployment	Tagging Date	Fish Total Length (cm)	Fish Girth (cm)	Video Duration (hh:mm)	Accelerometer Sampling Frequency (Hz) ^1^	Approximate Tag Retention Duration (h)
Fish 1	30/03/2020	135.5	99.2	10:08	50	68.00
Fish 2	10/06/2020	189.0	130.5	09:58	50	70.50
Fish 3	30/06/2020	161.0	107.8	02:49	50	70.25
Fish 4	10/07/2020	139.0	94.8	10:30	200	76.00
Fish 5	17/07/2020	140.0	99.2	10:30	200	70.50
Fish 6	29/07/2020	189.0	124.4	10:00	200	56.00

^1^ The sampling frequency for the magnetometer and gyroscope was 50 Hz for all deployments.

**Table 2:** Description of behavioral classes used to label the inertial measurement unit data. See Supplementary Materials Video S1 for examples of each behavior.

Behavior	Description
Backward Swimming	Reversing motion that occurs by undulating the pectoral fins.
Boom	Low-frequency single-pulse sound.
Gulping	Quick mouth movement that does not produce sound.
Burst Swimming	Fast forward movement, usually in response to a stimulus.
Feeding	Consumption of a prey item.
Forward Swimming	Forward movement that results in side-to-side swaying of the tag, reflecting the gait and tail-beat of the animal.
Gliding	Forward movement that does not result in swaying of the tag.
Hovering	Occurs when the animal appears largely motionless in the water column (rather than resting on substrate). May include small movements/adjustments.
Turning	A change in direction.
Listing	Less exaggerated than rolling. Animal rotates on its longitudinal axis to an angle <45°.
Resting	Animal appears to sit motionless on the substrate.
Rolling	Animal rotates on its longitudinal axis to an angle greater than 45°. This behavior may involve the individual full inverting its body so the dorsal surface makes contact with the substrate.
Shaking	Vigorous side-to-side movement. Often accompanies a boom or occurs during interactions with conspecifics.

**Table 3:** Number of observations contributed to each behavior class by each fish, and overall. Not all classes were included in the classifiers.

Behavior	Fish 1	Fish 2	Fish 3	Fish 4	Fish 5	Fish 6	Total
Backward Swimming	1344	312	25	393	-	57	2131
Boom	26	136	11	10	101	31	315
Gulping	45	26	16	136	33	3	259
Burst Swimming *	107	3	-	-	227	-	337
Feeding *	-	-	-	58	-	-	58
Forward Swimming	5501	5631	1577	6716	26,277	2313	48,015
Gliding *	339	6	-	-	-	-	345
Hovering	20,325	6750	7722	29,869	7846	32,663	105,175
Turning	176	285	183	22	2026	-	2692
Listing	58	72	6	53	157	37	383
Resting	6368	21,648	365	5	473	82	28,941
Rolling *	3	8	-	9	39	11	70
Shaking	190	589	121	542	155	415	2012

* Indicates classes omitted from classification.

**Table 4:** Kappa results for the conventional machine learning approaches: support vector machine (SVM) and random forest (RF), and the deep learning approach: convolutional neural network (CNN). Overall Performance for Kappa was calculated using Equations (13)–(15).

	Kappa		
Behavior	SVM	RF	CNN
Resting	0.8555	0.8414	0.8450
Hovering	0.8030	0.7927	0.7938
Forward Swimming	0.7889	0.8032	0.8121
Backward Swimming	0.8022	0.7971	0.8587
Boom	0.9114	0.8798	0.8014
Shaking	0.8566	0.8645	0.7508
Listing	0.7580	0.7589	0.8693
Turning	0.8450	0.8317	0.4480
Gulping	0.4512	0.4511	0.8293
Overall Performance	0.2097	0.5996	0.8331

**Table 5:** Sensitivity results for the conventional machine learning approaches: support vector machine (SVM) and random forest (RF), and the deep learning approach: convolutional neural network (CNN).

	Sensitivity		
Behavior	SVM	RF	CNN
Resting	0.6733	0.8640	0.9262
Hovering	0.8673	0.9078	0.9443
Forward Swimming	0.8251	0.7631	0.8007
Backward Swimming	0.6905	0.9785	0.9945
Boom	0.3282	0.8733	1.0000
Shaking	0.6355	0.8472	1.0000
Listing	0.7494	0.9822	0.9922
Turning	0.6032	0.9668	1.0000
Gulping	0.8961	0.9682	1.0000
Overall Performance	0.6965	0.9057	0.9620

**Table 6:** Specificity results for the conventional machine learning approaches: support vector machine (SVM) and random forest (RF), and the deep learning approach: convolutional neural network (CNN).

	Specificity		
Behavior	SVM	RF	CNN
Resting	0.9929	0.9947	0.9985
Hovering	0.9884	0.9873	0.9895
Forward Swimming	0.9633	0.9772	0.9941
Backward Swimming	0.9619	0.9958	0.9936
Boom	0.9961	0.9917	0.9993
Shaking	0.9579	0.9857	0.9989
Listing	0.9581	0.9946	0.9980
Turning	0.9699	0.9967	0.9906
Gulping	0.9194	0.9865	0.9996
Overall Performance	0.9675	0.9900	0.9958

**Table 7:** *F*_1_-score results for the conventional machine learning approaches: support vector machine (SVM) and random forest (RF), and the deep learning approach: convolutional neural network (CNN).

	*F*_1_-Score		
Behavior	SVM	RF	CNN
Resting	0.7689	0.8988	0.9531
Hovering	0.8802	0.9002	0.9273
Forward Swimming	0.7666	0.7779	0.8644
Backward Swimming	0.6805	0.9708	0.9550
Boom	0.4743	0.8722	0.9967
Shaking	0.5708	0.8280	0.9960
Listing	0.7314	0.9717	0.9815
Turning	0.6228	0.9656	0.9877
Gulping	0.8504	0.9665	0.9976
Overall Performance	0.7051	0.9057	0.9621

**Table 8:** Matthews Correlation Coefficient results for the conventional machine learning approaches: support vector machine (SVM) and random forest (RF), and the deep learning approach: convolutional neural network (CNN).

	Matthews Correlation Coefficient		
Behavior	SVM	RF	CNN
Resting	0.7600	0.8909	0.9497
Hovering	0.8671	0.8885	0.9191
Forward Swimming	0.7407	0.7532	0.8535
Backward Swimming	0.6441	0.9676	0.9524
Boom	0.5127	0.8639	0.9963
Shaking	0.5401	0.8155	0.9954
Listing	0.6931	0.9679	0.9803
Turning	0.5904	0.9624	0.9831
Gulping	0.7913	0.9537	0.9974
Overall Performance	0.6821	0.8959	0.9586

## Data Availability

The data will be made available upon request to the authors.

## Supplementary Materials

The following are available online at https://www.mdpi.com/article/10.3390/s21196392/s1, Video S1: Tag video examples of each behavioral class.
